# Evidence of *Plasmodium vivax* circulation in western and eastern regions of Senegal: implications for malaria control

**DOI:** 10.1186/s12936-024-04932-z

**Published:** 2024-05-16

**Authors:** Aida S. Badiane, Bassirou Ngom, Tolla Ndiaye, Deirdre Cunningham, James Campbell, Amy Gaye, Aita Sène, Mouhamad Sy, Daouda Ndiaye, Davis Nwakanma, Jean Langhorne

**Affiliations:** 1 Laboratory of Parasitology and Mycology, Faculty of Medicine, Pharmacy and Odontology, Université Cheikh Anta Diop of Dakar, Darkar, Sénégal; 2 Centre International de Recherche et de Formation en Génomique Appliquée et de Surveillance Sanitaire (CIGASS), Dakar, Sénégal; 3 Malaria Immunology Laboratory, The Francis Crick Institute, 1 Midland Road, London, NW1 1AT UK; 4 Bioinformatics and Biostatistics Science Technology Platforms (STP), The Francis Crick Institute, 1 Midland Road, London, NW1 1AT UK; 5 Medical Research Council Unit The Gambia at London, School of Hygiene and Tropical Medicine, P.O Box 273, Banjul, The Gambia

**Keywords:** *Plasmodium vivax*, Diagnosis, Targeted sequencing, Senegal

## Abstract

**Background:**

Malaria elimination in Senegal requires accurate diagnosis of all *Plasmodium* species. *Plasmodium falciparum* is the most prevalent species in Senegal, although *Plasmodium malariae*, *Plasmodium ovale,* and recently *Plasmodium vivax* have also been reported. Nonetheless, most malaria control tools, such as Histidine Rich Protein 2 rapid diagnosis test (PfHRP2-RDT,) can only diagnose *P. falciparum*. Thus, PfHRP2-RDT misses non-falciparum species and *P. falciparum* infections that fall below the limit of detection. These limitations can be addressed using highly sensitive Next Generation Sequencing (NGS). This study assesses the burden of the four different *Plasmodium* species in western and eastern regions of Senegal using targeted PCR amplicon sequencing.

**Methods:**

Three thousand samples from symptomatic and asymptomatic individuals in 2021 from three sites in Senegal (Sessene, Diourbel region; Parcelles Assainies, Kaolack region; Gabou, Tambacounda region) were collected. All samples were tested using PfHRP2-RDT and photoinduced electron transfer polymerase chain reaction (PET-PCR), which detects all *Plasmodium* species. Targeted sequencing of the nuclear 18S rRNA and the mitochondrial cytochrome B genes was performed on PET-PCR positive samples.

**Results:**

Malaria prevalence by PfHRP2-RDT showed 9.4% (94/1000) and 0.2% (2/1000) in Diourbel (DBL) and Kaolack (KL), respectively. In Tambacounda (TAM) patients who had malaria symptoms and had a negative PfHRP2-RDT were enrolled. The PET-PCR had a positivity rate of 23.5% (295/1255) overall. The PET-PCR positivity rate was 37.6%, 12.3%, and 22.8% in Diourbel, Kaolack, and Tambacounda, respectively. Successful sequencing of 121/295 positive samples detected *P. falciparum (*93%), *P. vivax (*2.6%), *P. malariae (*4.4%), and *P. ovale wallikeri* (0.9%). *Plasmodium vivax* was co-identified with *P. falciparum* in thirteen samples. Sequencing also detected two PfHRP2-RDT-negative mono-infections of *P. vivax* in Tambacounda and Kaolack.

**Conclusion:**

The findings demonstrate the circulation of *P. vivax* in western and eastern Senegal, highlighting the need for improved malaria control strategies and accurate diagnostic tools to better understand the prevalence of non-falciparum species countrywide.

**Supplementary Information:**

The online version contains supplementary material available at 10.1186/s12936-024-04932-z.

## Background

Malaria surveillance has been integrated as a core intervention in all malaria-endemic countries by the World Health Organization (WHO) since 2018 [1]. Senegal has made substantial efforts in malaria control through the National Malaria Control Programme (NMCP) using surveillance data to implement control strategies. Most detection strategies for malaria surveillance in peripheral health facilities (health posts) where most cases are diagnosed use *Plasmodium falciparum* Histidine Rich Protein 2 rapid diagnosis tests (PfHRP2-RDTs), which are specific for *P. falciparum*. However, the PfHRP2-RDT tests could miss low density *Plasmodium* infections and non-falciparum species usually responsible for asymptomatic infection [2]. Similarly, home-based management of malaria in high-transmission areas of the country only targets symptomatic individuals diagnosed by PfHRP2-RDTs. These interventions do not capture these types of infections in the health facilities and the communities leading to inappropriate strategies.

For elimination and eradication purposes, there is a need for accurate diagnosis of all *Plasmodium* species. Although *P. falciparum* is the most prevalent species in sub Saharan Africa, *Plasmodium malariae*, *Plasmodium ovale (P. ovale curtisi* and *P. ovale wallikeri)* and recently *Plasmodium vivax* have been reported [3, 4]. *Plasmodium vivax* and *P. ovale* have hypnozoites that could be responsible for relapses years after the first infection [5, 6] and *P. malaria*e presents chronic infection with long parasite carriage. This chronicity requires complementary strategies to detect and treat these infections, thus the real burden of the non-falciparum species needs to be assessed. This could be achieved by using appropriate tools and strategies for better detection and identification of all *Plasmodium* spp responsible for infection.

Using highly sensitive tools such as Next Generation Sequencing (NGS) could overcome the limit of detection of standard malaria diagnosis tools that can miss low density parasitaemia which is often the case in asymptomatic malaria infection at the community level. Sequencing allows the detection of both *P. falciparum* and non-falciparum species for all levels of parasitaemia and to capture additional cases that are missed by the current surveillance system. The availability of more accurate data could help reveal the true burden of malaria and inform the adjustment of interventions for malaria control, elimination, and ultimately eradication.

Here, a targeted sequencing approach was used to amplify regions of two genes, the small subunit ribosomal gene of the 18S rRNA (*ssu*) and the mitochondrial cytochrome B (*cytb*). This enabled assessment of the burden of *Plasmodium* species among asymptomatic and symptomatic subjects in the community and febrile individuals with a negative HRP2-based RDT in western and eastern areas in Senegal during the transmission season of 2020–2021.

## Methods

### Study sites

Patients were recruited during the malaria transmission season of 2020–2021 in three sites across Senegal; Diourbel located in the West, Kaolack in the west central and Tambacounda in the East of Senegal. In Diourbel the participants were recruited in the areas of Sessène, Kaolack in Parcelles Assainies, and Tambacounda in the site of Gabou situated in the department of Bakel. According to 2021 data of the National Malaria Control Programme (NMCP), malaria prevalence was intermediate in Diourbel and high in Kaolack and Tambacounda. In Diourbel and Kaolack the peak of malaria transmission starts in October/November and continues until January/February. In 2021 malaria incidence was reported to be 147, 28 and 9 cases per 1000 inhabitants in Tambacounda, Kaolack and Diourbel, respectively. Diourbel has a desert climate with an average temperature of 28.9 °C and average rainfall of 516 mm. In Kaolack, the average temperature is 28.6 °C and the average rainfall is 678.5 mm. In Tambacounda, rainfall totals 735 mm per year and the average temperature is 29.4 °C (Météo Senegal,) [7–9].

### Patient recruitment and sample collection

In Diourbel and Kaolack a community recruitment was conducted and included symptomatic and asymptomatic individuals of all ages. In Tambacounda, febrile patients who had a negative *P. falciparum* HRP2-based rapid diagnosis test were included in the study. Sample collection was carried out in December 2020 in Tambacounda and in January 2021 in Diourbel and Kaolack. For each participant, a RDT was performed, and blood was spotted on Whatman filter paper and dried at room temperature. Epidemiological data were recorded, including age, sex, address, symptoms, and history of recent travel.

### Plasmodium infection/malaria diagnosis

*Plasmodium* infection was assessed during enrolment using HRP2-based RDT (SD BIOLINE Malaria P. falciparum Ag Test/HRP-II). Further, a Photoinduced Electron Transfer PCR (PET-PCR), and a nested PCR were performed. Parasite DNA was extracted from filter paper samples using QIAamp DNA Mini kit (Qiagen, QIAGEN, USA) according to the manufacturer’s instructions. Molecular identification of *Plasmodium* species was carried out using the photo-induced electron transfer (PET)-PCR assay [10] on an Applied Biosystems™ 7500 Fast Dx Real-Time PCR Instrument (Applied BioSystems). For each experimental run both a negative (no template) and a positive control sample for each species were included. *Plasmodium* genus and *P. falciparum* PET-PCR were performed in a multiplex format as previously described [10] and the other species, *P. malariae*, *P. vivax*, and *P. ovale* PET-PCR reactions were run separately. All PCR reactions were conducted in a 20 μl reaction mixture containing, 5 μl of template DNA, 10 μl of 2× TaqMan Environmental Master-Mix 2.0 (Applied BioSystems), and 250 μM of each forward and reverse primers of each species. Cycling conditions were as follows: initial hot start at 95 °C for 15 min, followed by 45 cycles of denaturation at 95 °C for 20 s and annealing at 60 °C for 40 s. Samples with a cycle threshold (CT) of 40 or less were considered as positive [10, 11].

### Amplicon sequencing

Positive samples with PET-PCR were sequenced with the NGS platform, positive controls for each species were also sequenced. The amplicon sequencing for species and genotype identification was performed for the small subunit ribosomal of the 18S rRNA gene (*ssu*) and the mitochondrial cytochrome b (*cytb*) using previously published primers [12].

All primers were 5′-fused to universal tail sequences. The amplification was carried out according to a previously published protocol [12], the amplicons from each gene were pooled for one sample. The amplicons were purified using SPRI select beads according to standard procedures (Beckman Coulter, Life sciences). Amplicon size and purity were determined using a Tapestation. Barcode Index and Illumina sequencing adapters were attached using The Nextera^®^ XT Index kit. The samples were then quantified, normalized and sequenced using an Illumina Miseq sequencer. Samples were sequenced in three pools as 2 × 250 bp paired-end reads.

### Analysis of amplicon sequencing reads

Paired-end reads in fastq format were assessed to remove failed samples. Samples with 75 percent of reads with lengths below 100 nt were excluded from subsequent analysis. Reads were mapped to a reference database containing sequences of the *ssu* and *cytb* gene for five *Plasmodium* spp obtained from Genbank (Additional file 1: Table S1), using the bwa-mem aligner [13].

**Fig. 1:**
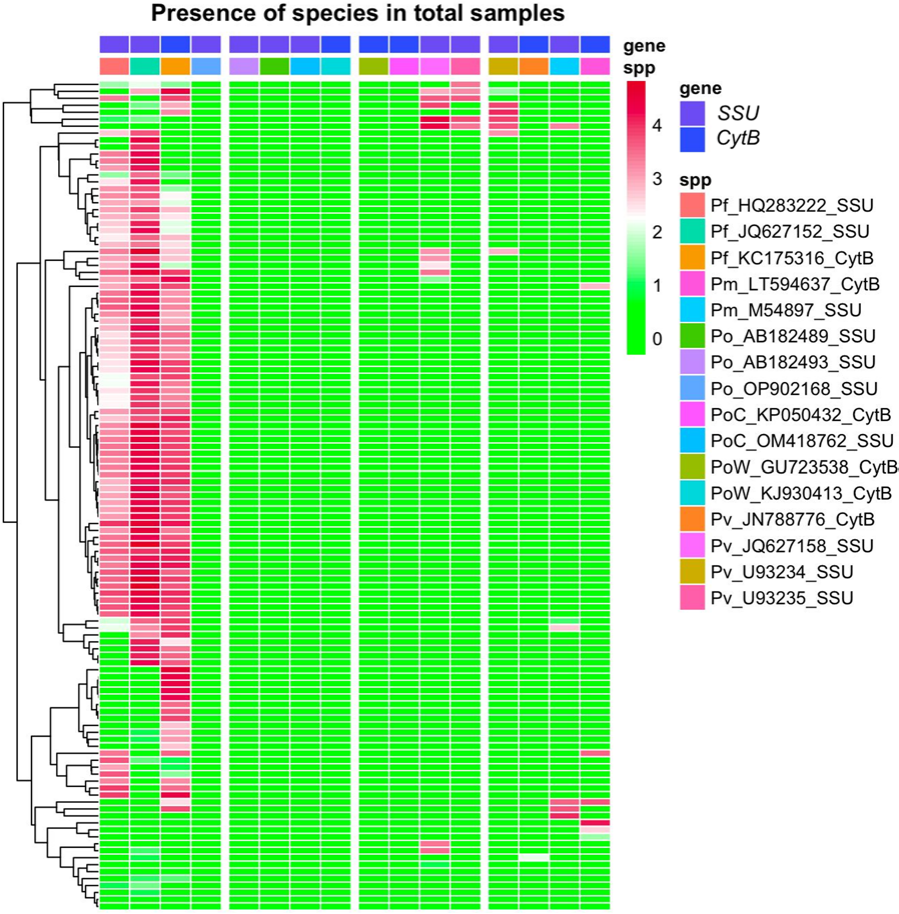
HYPERLINK "sps:id::fig1||locator::gr1||MediaObject::0" Heatmap summarizing the number of reads mapping to each reference sequence (columns) per sample (rows). Read counts were transformed to log10 pseudo-counts (read count + 0.5). Larger read counts imply stronger evidence of an identification. Pv: *Plasmodium vivax*, Pm: *Plasmodium malariae*, Pf: *Plasmodium falciparum*, Po: *Plasmodium ovale*, PoC: *Plasmodium ovale curtisi*, PoW: *Plasmodium ovale wallikeri*

Mapped reads with five or more soft-clipped bases were removed from the alignments using samclip (https://github.com/tseemann/samclip) as were reads with greater than four mismatches to the reference sequences. Finally, high-quality alignments (MAPQ of 60+) were counted for each sample and a positive identification of a species was recorded where at least 10 reads for any amplicon were identified (Fig. 1).

## Results

### *Plasmodium* genus infection characteristics

During the malaria transmission season in 2020 a total of 3000 samples were collected in three different sites Kaolack, Diourbel and Gabou towards the end of malaria transmission season. In the Tambacounda region, 1000 patients with a negative RDT were recruited. In the Kaolack and Diourbel regions, 1000 consenting individuals (247 had symptoms; 8 in Diourbel and 239 in Kaolack) have been recruited in the Sessene and Parcelles Assainies neighbourhoods, respectively.

The PfHRP2-based RDT had a positivity of 4.8% overall; 9.4% (94/1000) and 0.2% (2/1000), respectively in Diourbel and Kaolack. In Tambacounda, all patients had a negative PfHRP2-based RDT.

**Fig. 2:**
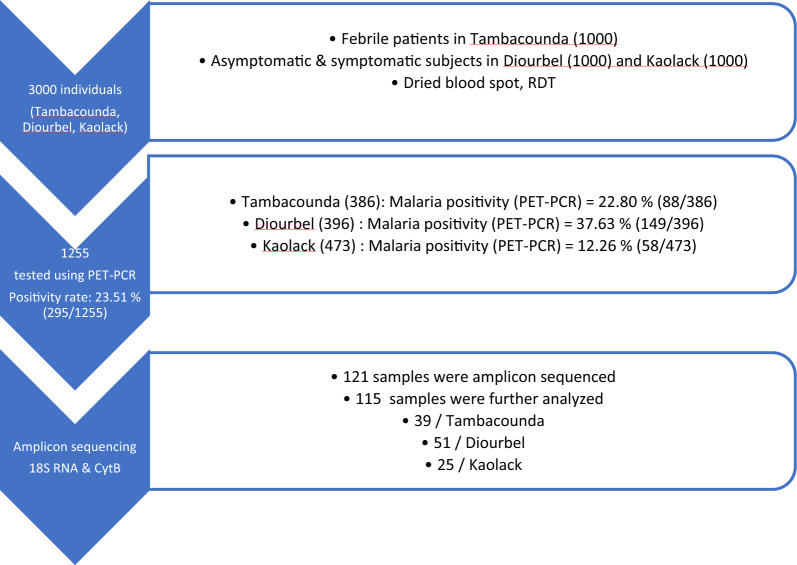
Flow chart of sample processing. From the 3000 samples collected; 396; 473 and 386, respectively from Diourbel, Kalolack and Tambacounda were tested with PET-PCR. 121 PET-PCR positive samples were sequenced

Among the 3000 samples collected, 396 from Diourbel, 473 from Kaolack and 386 from Tambacounda were randomly selected and a PET PCR for *Plasmodium* spp was performed (Fig. 2). With the PET-PCR, a positivity rate of 23.51% (295/1255) was found. According to the PET-PCR, *Plasmodium* spp infection in Diourbel (Sessene) and Kaolack (Parcelles Assainies) was 37.63% (149/396) and 12.26% (58/473), respectively. In Tambacounda (Gabou) where febrile patients with PfHRP2-RDT negative were recruited, malaria positivity was 22.80% (88/386) (Fig. 2).

### *Plasmodium* species identification

**Fig. 3:**
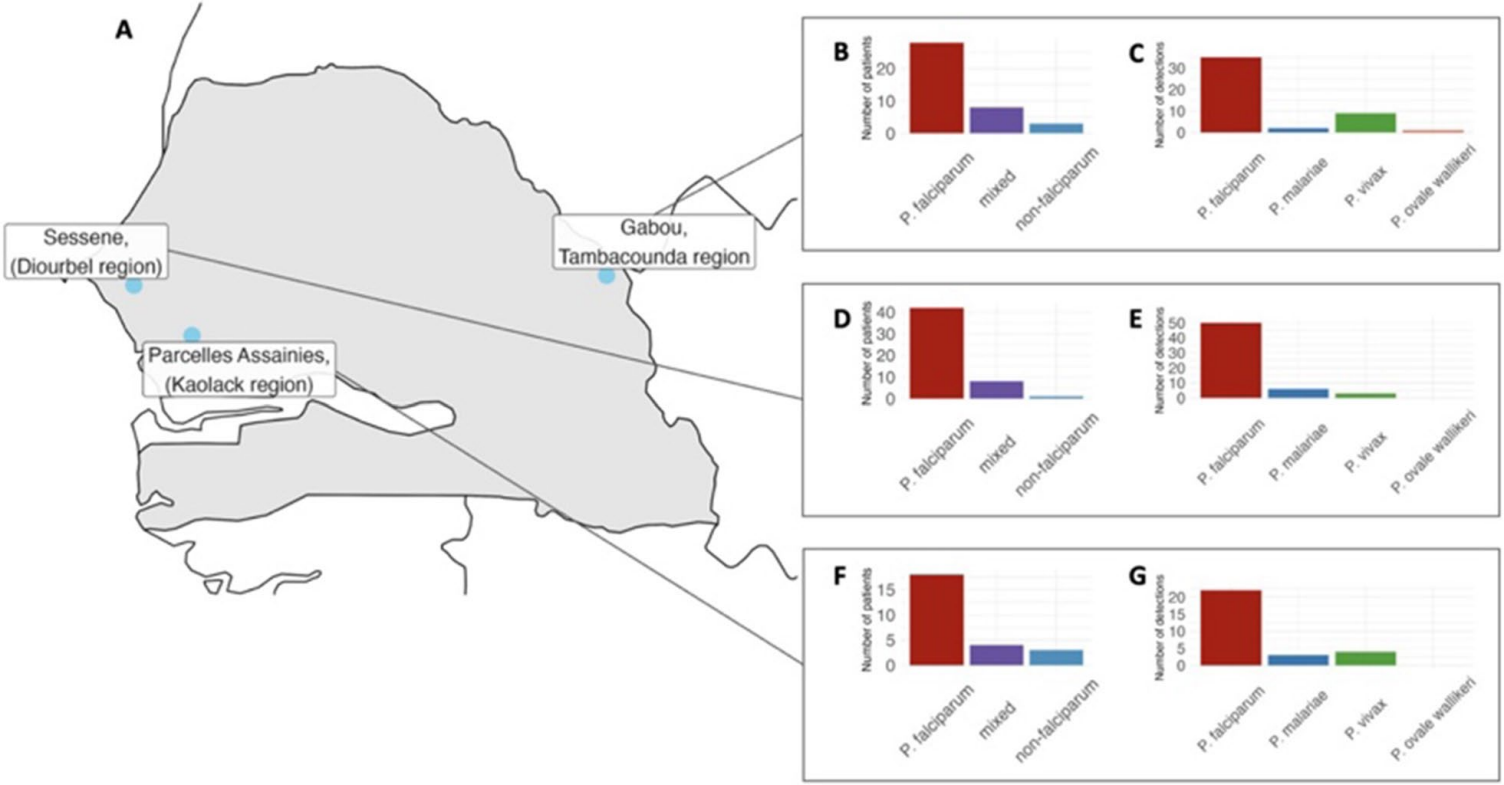
Infections with *Plasmodium* species across three sites in Senegal. **A** Map of Senegal (area in grey) showing the location of three sites where samples were collected (blue dots). **B** Barplot summarising the number of patients in the Tambacounda (Gabou) region that were found to be infected with either *P. falciparum* alone, a mixture of *P. falciparum* and another species (mixed) or with *Plasmodium* species other than *falciparum* (non-*falciparum*). **C** Barplot summarising the total number of detections of *Plasmodium* species in patient samples collected in the Tambacounda region. **D** Barplot summarizing the number of patients in the Diourbel region with single or mixed *Plasmodium* infections, details as for panel B. **E** Barplot summarizing the total number of species detected in samples collected from the Diourbel region, details as for panel **C**. **F** Barplot summarising the number of patients in the Kaolack region with single or mixed *Plasmodium* infections, details as for panel **B**. **G** Barplot summarizing the total number of species detected in samples collected from the Kaolack region, details as for panel **C**

Species identification was performed using amplicon deep sequencing of the *ssu* of the 18S rRNA (nucleus) and the *cytb* (mitochondrion) genes. A total of 121 samples were sequenced; six samples were excluded due to poor quality sequencing data. The remaining 115 samples were further analysed. Of these 115 samples, 51 were taken from Diourbel, 39 from Tambacounda and 25 from Kaolack (Fig. 2). NGS amplicon sequencing identified all four *Plasmodium* species: *P. falciparum*, *P. vivax*, *P. malariae* and *P. ovale wallikeri*. *P. falciparum* represented respectively 44% (51/115), 22% (25/115) and 34% of the infections in Diourbel, Kaolack and Tambacounda (Fig. 3, Additional file 1: Table S2). Non-falciparum infections (mono and mixed infections) were 23.5% (27/115) overall; 7.8% (9/115) in Diourbel; 6.1% (7/115) in Kaolack and 9.6% (11/115) in Tambacounda. Non-falciparum mono-infections represented 6.1% (7/115) of the infections. *Plasmodium malariae* represented 3.5% (4/115) of mono-infection, *P. vivax* 2.6% (3/115) and *P. ovale wallikeri* 0.836% (1/115) (Additional file 1: Table S2). Mixed infections represented 17.39% (20/115) overall; 6.97% (8/115); 3.48% (4/115) and 6.97% (8/115), respectively, in Diourbel, Kaolack and Tambacounda (Fig. 3, Additional file 1: Table S2).

In terms of identification, some infections were identified by either the *ssu* or the *cytb* gene. Among the eight non-falciparum mono-infections, three were identified with the *ssu* gene and five with the *cytb* (Additional file 1: Table S3).

**Fig. 4:**
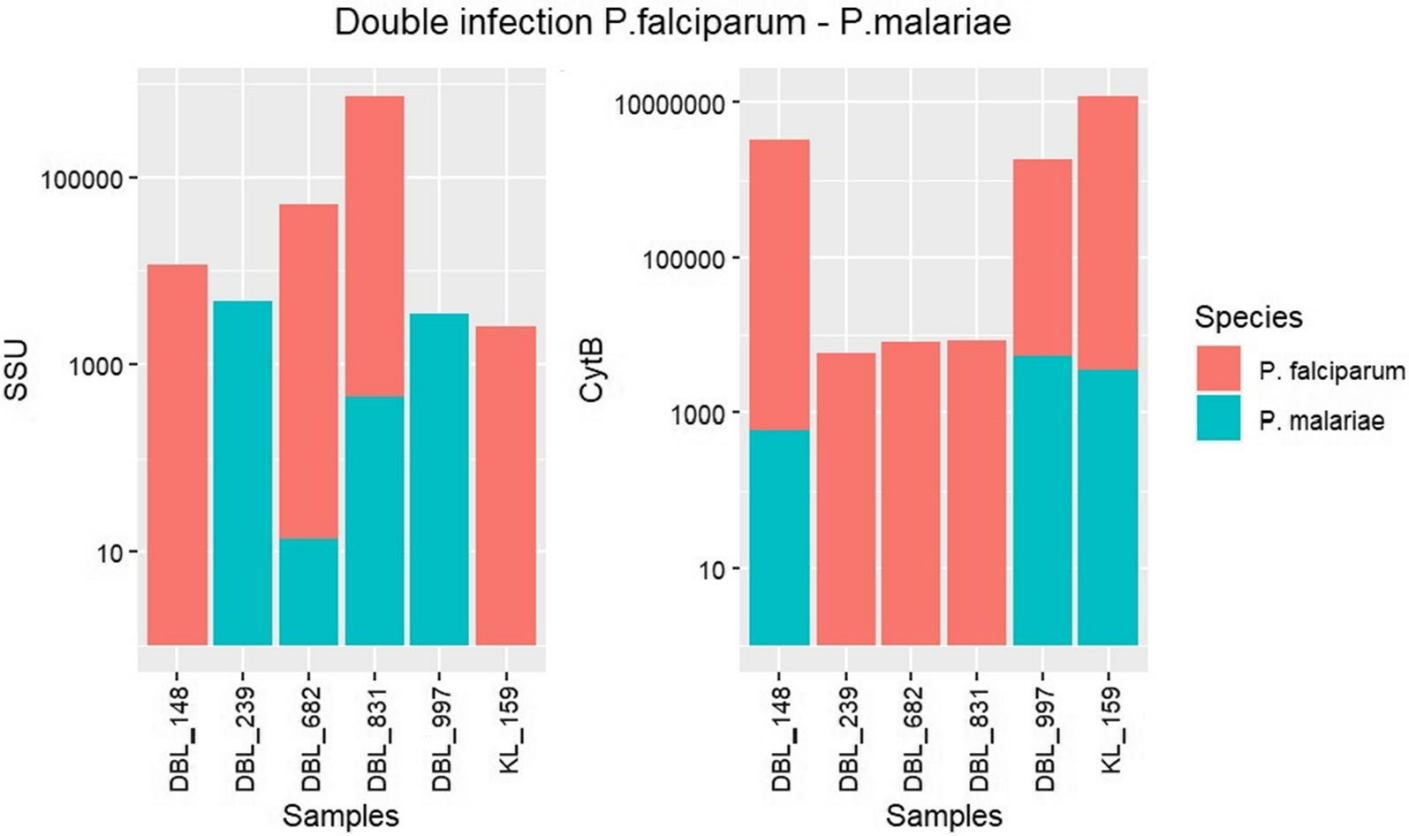
Barplot summarizing the number of *ssu* and *cytb* reads aligned to *P. falciparum* and *P. malariae ssu* and *cytb* genes in samples with mixed infections. Green represents the number of reads for *P. malariae*, and red the number of reads for *P. falciparum*. The association is represented by a bar with two colours (green and red). Due to the plot low number of reads for some samples (DBL_148; DBL_239; KL_159; DBL_682; DBL_831) the association is not shown in the bars

For the mixed infection of *P. falciparum* and *P. malariae*, the *ssu* gene failed to identify two *P. malariae* infections (DL_148 and KL_159) which were identified with the *cytb* gene and for DBL_682 the number of reads for the *cytb* was very low (14 reads). The *cytb* gene could not identify three *P. malariae* infections (Fig. 4, Additional file 1: Tables S4 and S6).

**Fig. 5:**
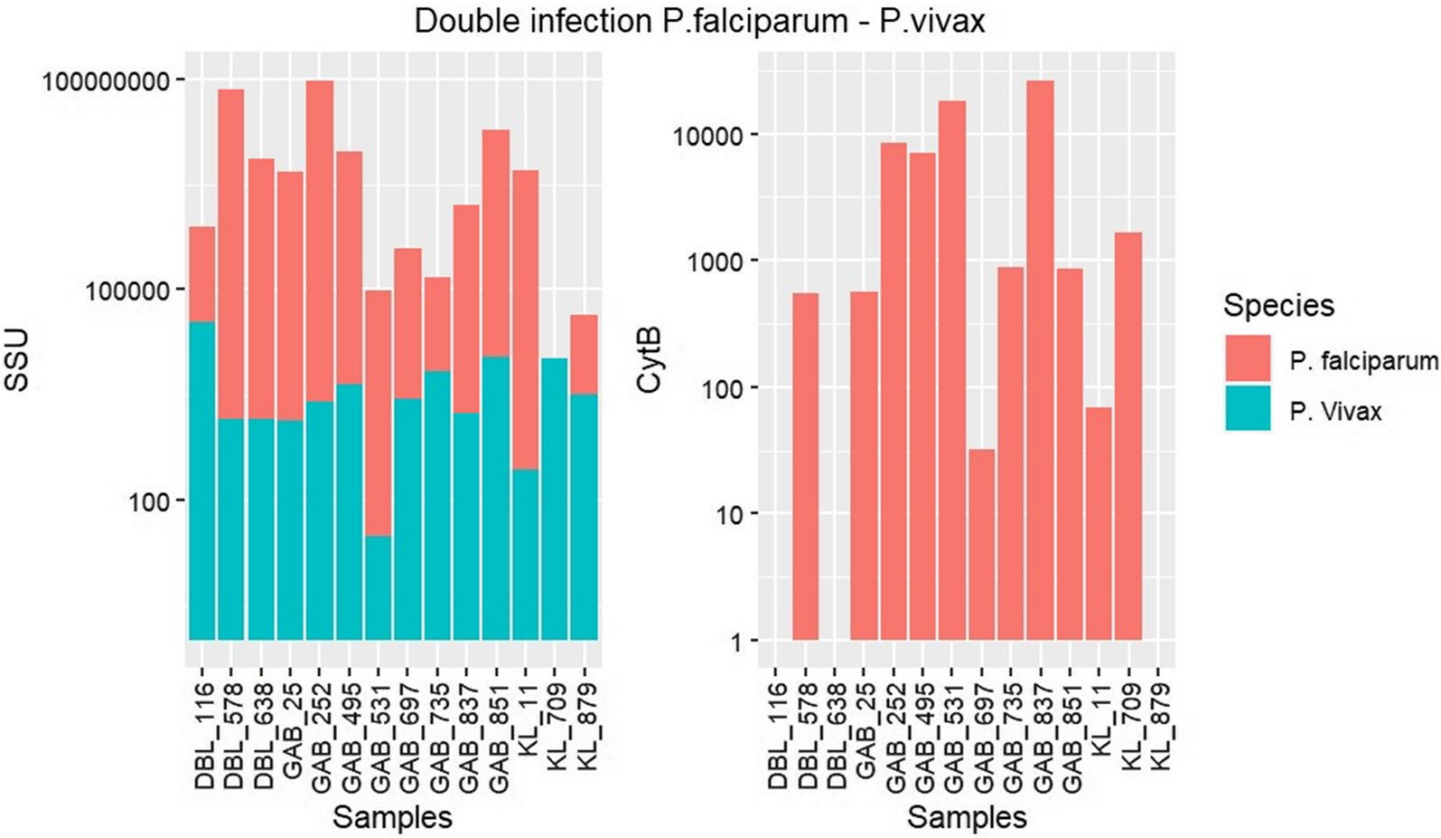
Barplot summarizing the number of reads aligned to *P. falciparum* and *P. vivax ssu* and *cytb* genes in samples with mixed infections. Green represents the number of reads for *P. vivax*, and red the number of reads for *P. falciparum*. The association is represented by a bar with two colours (green and red). Due to the low number of reads for some samples the *cytb* gene, the association is not shown in the bars

In mixed infections Pf/Pv; *P. vivax* was identified only with the *ssu* gene; three P*. falciparum* infections could not be detected using the *cytb* gene. Only one *P. falciparum* infection was not detected with the *ssu* gene (Fig. 5, Additional file 1: Tables S5 and S6).

### Distribution of* Plasmodium* variants and species

As shown in Fig. 3, *P. falciparum* was responsible for 93.0% (mono- and mixed infections with other species) of the infections among which mono-infections represented 76.5% (88/115) and distributed as follows; 37.5% (42/115) in Diourbel, 27.0% in Tambacounda (31/115) and 18.3% in Kaolack (21/115). Multi-infections of *P. falciparum* with other species represented 16.5% (19/115). *Plasmodium falciparum* was present, respectively with *P. malariae* (Pf/Pm) and *P. vivax* (Pf/Pv) in 5.2% (6/115) and 11.3% (13/115) of the infections. No co-infection with *P. ovale* was found.

**Fig. 6:**
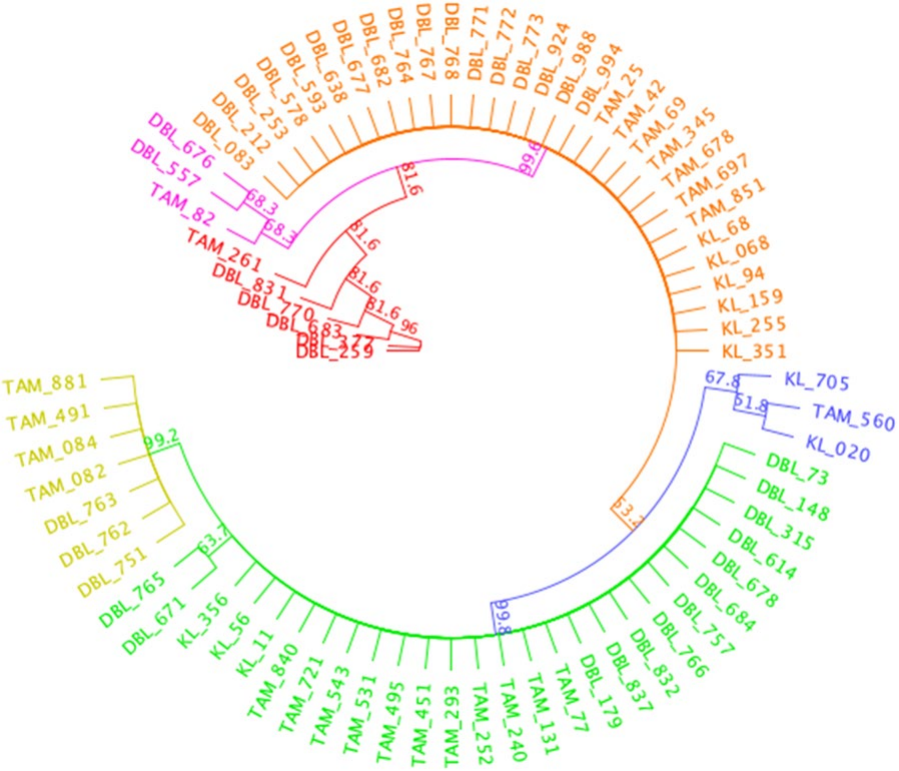
Phylogenetic tree comparing *P. falciparum* 18S rRNA gene sequences. *P. falciparum* strains are divided into 6 branches represented by different colours. The green branch comprises twenty-seven (27) variants; thirteen (13) from Diourbel, eleven (11) from Tambacounda and only three (03) variants from Kaolack. DBL, KL, TAM associated with numbers represent sample identifiers

Sequencing of the *ssu* gene of *P. falciparum* was used to determine the extent of strain variation across the samples. This analysis showed six groups (Fig. 6). The green and orange groups contain the highest number of *P. falciparum* variants. The orange group contains variants from the three study sites: Diourbel (17), Kaolack (06) and Tambacounda (07). The green group is composed of 13 from Diourbel; 11 from Tambacounda and three from Kaolack. The yellow group is represented by Diourbel (03) and Tambacounda (04). The Red group is composed of five variants from Diourbel and one from Tambacounda. The pink group contains three strains: two from Diourbel and one from Tambacounda (Fig. 6). The nucleotide sequences that differentiate the groups are presented in Additional file 1: Table S6. The groups are distributed across all sites; no variant is specific to one site.

*Plasmodium vivax* was responsible for 13.04% (15/115) of the infections; 11.30% (13/115) polyinfections and 1.74% (2/115) mono-infections (Additional file 1: Table S2). The prevalence of *P. vivax* was 6.96% (8/115) in Tambacounda; 3.48% (4/115) in Kaolack and 2.61% (3/115) in Diourbel. Mono-infection was found once in Tambacounda and Kaolack. *Plasmodium malariae* and *P. vivax* were associated in 1 sample and identified with the *ssu* gene, and the patient was a 26-year-old female from Tambacounda. The number of reads for the *ssu* gene was 25,098 for *P. vivax* and 1884 for *P. malariae*.

Two of the three individuals infected with *P. vivax* presented symptoms of headaches and were from Kaolack and Tambacounda. All mixed infections with *P. falciparum* presented symptoms except four (03 from Diourbel and 01 from Kaolack).

**Fig. 7:**
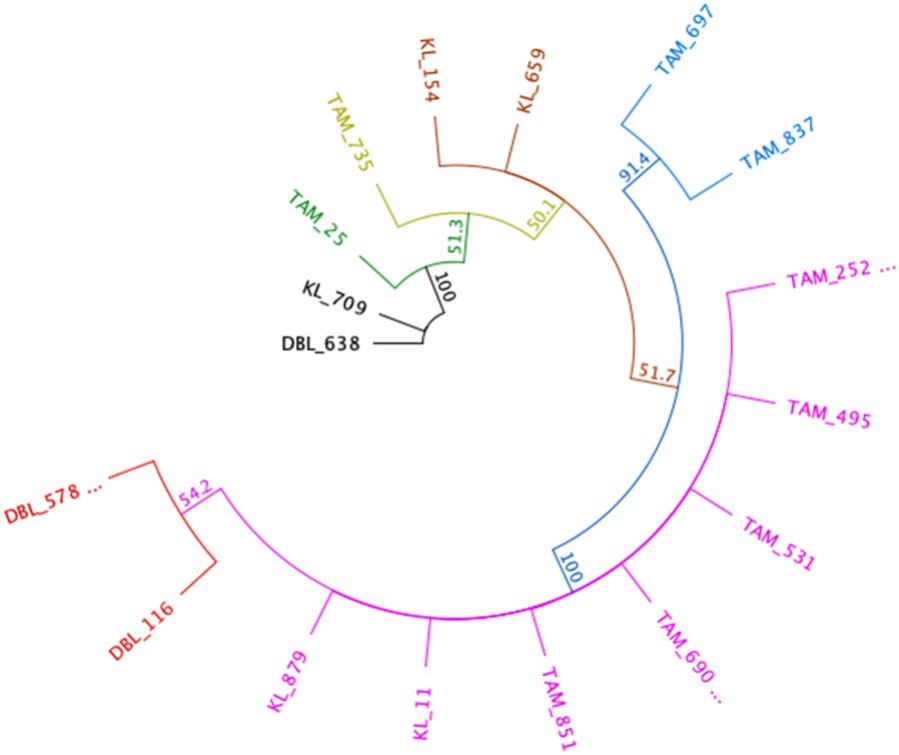
Phylogenetic tree comparing *P. vivax* 18S rRNA gene sequences. *P. vivax* strains are divided into seven branches, each represented by a different colour. The violet branch contains more variants, with seven (07) from Tambacounda and two (02) from Kaolack. The other branches are made up of two variants, and two branches have only one variant. DBL, KL, TAM associated with numbers represent sample identifiers

The phylogenetic tree of *P. vivax* derived from the *ssu* gene is composed of seven (07) branches (Fig. 7). The nucleotides differentiating the strains are listed in Additional file 1: Table S6. The black branch includes two strains from Diourbel and Kaolack. The green and the yellow branches include each one variant from Tambacounda. The orange, red and blue branches contain each two variants; respectively from Kaolack, Diourbel and Tambacounda. The purple branch consists of seven variants: five from Tambacounda and two from Kaolack (Additional file 1: Table S6).

*Plasmodium malariae* was responsible for 9.56% of the infections (11/115), among which 3.47% (4/115) were mono-infections and 6.09% (0/115) were poly-infections (Additional file 1: Table S2). *Plasmodium malariae* prevalence in Diourbel was 5.22% (6/115); only one infection was a mono-infection. In Kaolack, *P. malariae* prevalence was 2.61% (3/115), two poly-infections and one mono-infection. In Tambacounda, *P. malariae* was responsible for two infections; one mono-infection and one associated with *P. vivax* (Fig. 3). There were three different variants of *P. malariae* among the samples. (Additional file 1: Table S6). *Plasmodium ovale wallikeri* was found in only one mono-infection in Tambacounda.

Infections with *P. malariae* mono and mixed infection Pf/Pm was found in both adults and children, characteristics of patients are presented in Additional file 1: Tables S3 and S4.

## Discussion

An accurate estimation of the prevalence of malaria and the causative species is needed to achieve its elimination. The aim of this study was to determine the frequency of *Plasmodium* species in different endemic sites in Senegal towards the end of the malaria transmission season. The study population included asymptomatic and symptomatic individuals sampled in the community in Diourbel (Sessene) and Kaolack (Parcelles Assainies) and symptomatic patients with a negative PfHRP2-RDT test at the health post in Tambacounda.

Using a genus-specific PET-PCR, that identifies all *Plasmodium* species, followed by targeted deep amplicon sequencing of the *ssu* and the *cytb* genes to distinguish the different species, we detected *P. falciparum*, *P. vivax*, and *P. malariae* in Senegal, with a rare detection of *P. ovale wallikeri*. However, the genes used to identify the *Plasmodium* species are not discriminative enough to allow for the detection of variants or the multiplicity of infections. Although circulation of *P. vivax* in southern and northern Senegal [14–18] has been debated, here, *P. vivax* was identified in eastern (Tambacounda, Bakel) and central (Diourbel and Kaolack) regions of Senegal. In sub-Saharan Africa several studies have reported *P. vivax,* including in countries bordering Senegal, e.g. Mali and Mauritania, [19–22]. Most *P. vivax* infections (7/13) in this study were identified in Tambacounda, where the samples were collected in the health post of Gabou (Bakel district) near the border with Kayes in Mali where *P. vivax* has been observed [23]. However, differences in patient recruitment methods between sites; community sampling from Kaolack and Diourbel and symptomatic sampling of patients in Tambacounda, do not allow a direct comparison of prevalence. However, this strategy includes two population groups likely to be infected by non-falciparum *Plasmodium* that are often not considered in NMCP diagnostic and surveillance strategies.

Despite the evidence of the circulation of *P. vivax* in sub-Saharan Africa, most control and elimination strategies focus on *P. falciparum*. *P. vivax* produces hypnozoites that cannot be detected using current diagnostic methods used in endemic areas nor treated with artemisinin-based combination therapy and, therefore, will maintain the transmission. Beside *P. vivax*, *P. malariae* was the third most prevalent species and only one infection with *P. ovale wallikeri* was identified. A few studies have reported the circulation of *P. malariae* and *P. ovale* in symptomatic individuals in Senegal, *P. malariae* being responsible for acute renal failure [17, 24–27]. It has been reported that *P. malariae* and *P. ovale* are responsible for severe and persistent cases of malaria [28, 29]. Thus, those undiagnosed, and untreated cases of malaria can develop into severe malaria, leading to hospitalization and possibly death.

The prevalence of non-f*alciparum* species was higher than expected both in symptomatic patients and asymptomatic individuals. With current control methods, there is a decrease in malaria caused by *P. falciparum,* and this could lead to an increase in malaria caused by other species that are neglected [30, 31]. This could be explained by the interaction of the species within the host as the presence of one species at a parasite density sufficient to trigger treatment seeking restricts the ability of previous lower density infections of the other species from persisting [31].

It has been shown that *P. falciparum* genetic diversity is a useful metric to estimate malaria transmission in endemic areas [30, 31]. In this study, the non-falciparum species identified also show a genetic diversity of the *ssu* gene suggesting a high frequency of transmission of these species in the areas studied. The genetic diversity is high in areas where transmission is high and low in regions implementing effective control strategies [32–36]. These results are based mainly on *P. falciparum,* but application of these approaches could be valuable for the non-*falciparum* species estimating the intensity of their transmission in endemic areas.

The positivity rate using PET-PCR was much higher than with RDTs for *Plasmodium* spp. infection, which is due to the higher sensitivity of the molecular techniques and to the detection of the non-falciparum species which were missed by PfHRP2-RDT. In Tambacounda, situated in the red zone where malaria transmission is the highest in the country, a high proportion of the infections was missed with the PfHRP2-RDT among febrile patients [37]. Guidelines for the biological diagnosis of malaria in Senegal are the use of PfHRP2-RDTs in health posts receiving most of malaria cases, as well as consultation departments [38]. Microscopy is available in health centres and hospital laboratories and the LAMP technique in used in the northern zone, which is in the process of eliminating malaria [38]. In Senegal, control strategies are directed against *P. falciparum* which is thought to be responsible for 99% of malaria cases based on data from health facilities and home-based management where symptomatic cases are diagnosed using PfHRP2-RDTs that detect only *P. falciparum*. In addition, the non-falciparum species may be more difficult to treat because of relapses [5, 6].

Current diagnosis strategy leads to an underestimation of non-falciparum species, which are reported in some health facilities using other diagnostic methods or during research studies. Thus, there is an urgent need to establish the prevalence of all species of *Plasmodium* circulating in the country to inform a better management of these undiagnosed malaria cases in health facilities that rely on only PfHRP2-RDTs for diagnosis. Thus, there is a need for sensitive diagnostic tools that can detect all *Plasmodium* species circulating in the country for a better management of malaria and for elimination purposes.

Asymptomatic *Plasmodium* infection should be investigated for determining the real burden and implement adequate strategies for elimination purposes. However, currently in Senegal no strategy targets the asymptomatic infections. In this study a high proportion of infections detected in individuals recruited in the community were asymptomatic. Most of these infections were not detected with the PfHRP2-RDT as they are often characterized by a low density of parasites that often cannot be detected by microscopy or by the RDTs used in endemic areas for malaria diagnosis [39, 40]. The prevalence of *Plasmodium* infection was much higher in Diourbel than Kaolack, which is in line with the NMCP data from 2021 malaria incidence [37]. These infections not seen in health facilities would, therefore, go undetected and untreated. In addition, asymptomatic low-density infections due to non-falciparum species, which go undiagnosed, could become chronic and produce gametocytes likely to infect mosquitoes and contribute to the maintenance of malaria transmission [40–42].

NMCP malaria stratification in Senegal is based on PfHRP2-RDTs positivity among febrile patients attending health facilities and symptomatic cases diagnosed with home-based malaria management by the home care provider. While this strategy is less costly it does not consider low density *Plasmodium* infection, asymptomatic cases, malaria caused by *P. falciparum* with HRP2/3 deletion or malaria due to non-falciparum species [43–45]. To overcome these challenges there is a need to include the screening of asymptomatic *Plasmodium* infections and the use of more sensitive diagnostic tools that can detect and identify all *Plasmodium* species circulating in the country.

### Supplementary Information

**Additional file 1: Table S1.** Reference sequences of *Plasmodium* species used for read mapping. **Table S2.** Parasites species composition by site. **Table S3.** *Non-falciparum* monoinfection distribution by site. **Table S4.** Characteristics of mixed *P. falciparum* and *P. malariae* infections. **Table S5.** Characteristics of mixed *P. falciparum* and *P. vivax* infections. **Table S6.** Nucleotide differences between *Plasmodium* species based on the 18S rRNA gene.

## Data Availability

All data generated or analysed during this study are included in this article and its Additional files.

## Abbreviations

PfHRP2-RDT: Histidine Rich Protein 2 rapid diagnosis test
NGS: Next Generation Sequencing
DBL: Diourbel
KL: Kaolack
TAM: Tambacounda
WHO: World Health Organization
NMCP: National Malaria Control Programme
*Ssu*: Small subunit ribosomal gene of the 18S rRNA
*Cytb*: Mitochondrial cytochrome *B*
PET-PCR: Photo-induced electron transfer polymerase chain reaction
