# Supplementary material for: Evidence of Plasmodium vivax circulation in western and eastern regions of Senegal: implications for malaria control
Source: Malar J. 2024 May 16;23:149. doi: 10.1186/s12936-024-04932-z (PMC11097470; doi:10.1186/s12936-024-04932-z)
Supplement: Supplementary file 1 — Additional file 1: Table S1. Reference sequences of Plasmodium species used for read mapping. Table S2. Parasites species composition by site. Table S3. Non-falciparum monoinfection distribution by site. Table S4. Characteristics of mixed P. falciparum and P. malariae infections. Table S5. Characteristics of mixed P. falciparum and P. vivax infections. Table S6. Nucleotide differences between Plasmodium species based on the 18S rRNA gene. [file 12936_2024_4932_MOESM1_ESM.docx]

**Table S1: Reference sequences of *Plasmodium* species used for read mapping**

| Plasmodium species | GenBank Acc | local spp. ID |
| --- | --- | --- |
| *Plasmodium falciparum* 18S ribosomal RNA gene (S‐type) | HQ283222.1 | Pf_HQ283222_SSU |
| *Plasmodium falciparum* 18S ribosomal RNA gene (A‐type) | JQ627152.1 | Pf_JQ627152_SSU |
| *Plasmodium vivax* 18S ribosomal RNA gene (A‐Type) | JQ627158.1 | Pv_JQ627158_SSU |
| *Plasmodium vivax* 18S ribosomal RNA gene (O‐Type) | U93235.1 | Pv_U93235_SSU |
| *Plasmodium vivax* 18S ribosomal RNA gene (S‐Type) | U93234.1 | Pv_U93234_SSU |
| *Plasmodium malariae* 18S ribosomal RNA gene | M54897.1 | Pm_M54897_SSU |
| *Plasmodium ovale* 18S ribosomal RNA gene | AB182493.1 | Po_AB182493_SSU |
| *Plasmodium ovale* 18S ribosomal RNA gene | AB182489.1 | Po_AB182489_SSU |
| *Plasmodium ovale* po-38-01 small subunit ribosomal RNA gene | OP902168.1 | Po_OP902168_SSU |
| *Plasmodium ovale* curtisi A.KH small subunit ribosomal RNA gene | OM418762.1 | PoC_OM418762_SSU |
| *Plasmodium ovale wallikeri* cytochrome b gene | KJ930413.1 | PoW_KJ930413_CytB |
| Plasmodium *ovale wallikeri isolate UKMRL09 cytochrome b gene* | GU723538.1 | PoW_GU723538_CytB |
| *Plasmodium ovale curtisi* cytochrome b gene | KP050432.1 | PoC_KP050432_CytB |
| *Plasmodium malariae* cytochrome b gene | LT594637k | Pm_LT594637_CytB |
| *Plasmodium vivax* cytochrome b gene | JN788776 | Pv_JN788776_CytB |
| *Plasmodium falciparum* cytochrome b gene | KC175316.1 | Pf_KC175316_CytB |

**Table S2: Parasites species composition by site**

| Species | Infection Type | Number of samples | | | |
| --- | --- | --- | --- | --- | --- |
|  |  | Diourbel  % (N) | Kaolack  % (N) | Tambacounda  % (N) | Total  % (N) |
| *P. falciparum* | Mono Infection | 37.52 (42) | 183  (18) | 27.00  (28) | 76.52 (88) |
| *P. malariae* | Mono Infection | 0.84  (1) | 1.74  (2) | 0.84  (1) | 3.47  (04) |
| *P. ovale wallikeri* | Mono Infection | 0 | 0 | 0.84  (1) | 0.84 (1) |
| *P. vivax* | Mono Infection | 0 | 0.84  (1) | 0.84  (1) | 2.6  (2) |
|  | All mono Infection | 37.4  (43) | 18,4 (21) | 27  (31) | 82.61 (95) |
| *P. falciparum*  *P. malariae* | Mixed Double Infection | 4.35  (5) | 0.84  (1) | 0  ( | 5.22  (06) |
| *P. falciparum*  *P. vivax* | Mixed Double Infection | 2.61  (3) | 2.61  (3) | 6.09  (07) | 11.3  (13) |
| *P. malariae,*  *P. vivax* | Mixed Double Infection | 0 | 0 | 0.84  (1) | 0.84 (01) |
|  | all Mixed infections | 7  (8) | 3.48  (4) | 6.97  (8) | 17.4 (20) |
| *P. malariae*  *P. vivax*  *P. ovale*  *wallikeri* | *Non-falciparum* infections (mono and mixed infections) | 7.83  (9) | 6.09  (7) | 9.56  (11) | 23.48 (27) |
| Total | | 44.4  (51) | 21.7 (25) | 33.9  (39) | 100 (115) |

Pf: *Plasmodium falciparum*; Pm: *Plasmodium malariae*;  Pv: *Plasmodium vivax*; Pow: *Plasmodium ovale wallikeri*. Pf, Pm: association of Pf and Pm. Pf, Pv: association of Pf and Pv

**Table S3: *Non-falciparum* monoinfection distribution by site**

**.**

| Sample IDs | Site | Species | *ssu* | *cytb* | Age | Sex | Symptoms |
| --- | --- | --- | --- | --- | --- | --- | --- |
| GAB_217 | Tambacounda | *P. vivax* | 0 | 134 | 64 | F | Headaches and cough |
| KL_154 | Kaolack | *P. vivax* | 2456 | 0 | 25 | F | headaches |
| KL_659 | Kaolack | *P. vivax* | 3844 | 0 | 13 | M | No symptoms |
| DBL_290 | Diourbel | *P. malariae* | 0 | 432 | 23 | M | No data |
| GAB_723 | Tambacounda | *P. malariae* | 11152 | 0 | 35 | F | headaches |
| KL_184 | Kaolack | *P. malariae* | 0 | 44 | 23 | F | No symptoms |
| KL_387 | Kaolack | *P. malariae* | 0 | 14136 | 20 | F | No symptoms |
| GAB_859 | Tambacounda | *P. ovale wallikeri* | 0 | 84 | 28 | F | Fever, headache, cough |

**Table S4: Characteristics of mixed *P. falciparum* and *P. malariae* infections**

| **Mixed infections *P. falciparum and P. malariae*** | | | | | | | | |
| --- | --- | --- | --- | --- | --- | --- | --- | --- |
| **Samples** | **Study sites** | ***P. falciparum ssu*** | ***P. falciparum* *cytb*** | ***P. malariae* *ssu*** | ***P. malariae* *cytb*** | **Age** | **Sex** | Symptoms |
| DBL_148 | Diourbel | 11816 | 5600 | 0 | 604 | 19 | M | No data |
| DBL_239 | Diourbel | 0 | 5750 | 4662 | 0 | 10 | M | No data |
| DBL_682 | Diourbel | 3636 | 8096 | 14 | 0 | 8 | 0 | No symptoms |
| DBL_831 | Diourbel | 1612 | 8464 | 452 | 0 | 16 | M | headaches |
| DBL_997 | Diourbel | 0 | 360 | 3482 | 5346 | 14 | M | No symptoms |
| KL_159 | Kaolack | 2538 | 3420 | 0 | 3546 | 13 | M | No symptoms |

**Table S5: Characteristics of mixed *P. falciparum* and *P. vivax* infections**

| **Mixed infections *P. falciparum* and *P. vivax*** | | | | | | | |
| --- | --- | --- | --- | --- | --- | --- | --- |
| **Samples** | **Study sites** | ***P. falciparum* *ssu*** | ***P. falciparum* *cytb*** | ***P. vivax* *ssu*** | **Age** | **Sex** | **Symptoms** |
| DBL_116 | Diourbel | 22 | 0 | 34798 | 18 | M | No symptoms |
| DBL_578 | Diourbel | 52308 | 554 | 1388 | 16 | M | No symptoms |
| DBL_638 | Diourbel | 5292 | 0 | 1388 | 18 | M | No symptoms |
| GAB_25 | Tambacounda | 3424 | 562 | 1366 | 64 | F | headaches |
| GAB_252 | Tambacounda | 37708 | 8460 | 2546 | 20 | M | Fever, headache, chills |
| GAB_531 | Tambacounda | 3274 | 17952 | 30 | 18 | M | Fever, headache |
| GAB_697 | Tambacounda | 138 | 32 | 2732 | 14 | M | Chilling, fever |
| GAB_735 | Tambacounda | 22 | 886 | 6816 | 35 | F | Fever, headache |
| GAB_837 | Tambacounda | 944 | 26438 | 1694 | 6 | F | Fever, headache, cough |
| GAB-495 | Tambacounda | 2144 | 7104 | 4354 | 48 | M | Urinary burning |
| KL_11 | Kaolack | 18662 | 68 | 272 | 9 | M | No symptoms |
| KL_709 | Kaolack | 0 | 1668 | 10582 | 18 | M | chills, cough, colds |
| KL_879 | Kaolack | 14 | 0 | 3108 | 11 | M | No symptoms |
| GAB_851 | Tambacounda | 1732 | 853 | 10825 | 18 | F | Abdominal pain, headache |

**Table S6: Nucleotide differences between *Plasmodium* species based on the 18S rRNA gene**

| ***Plasmodium falciparum* (18S rRNA gene)** | | | | | | | | | | | | | | ***Plasmodium malariae* (18S rRNA gene)** | | | |
| --- | --- | --- | --- | --- | --- | --- | --- | --- | --- | --- | --- | --- | --- | --- | --- | --- | --- |
|  | Position | 345 | 139 to 141 | 144-145 | 147, 178, 215 | 149-150 | 153 | 155 | 157 to 174 | 175 -176 | 181, 185 | 189 to 191 | 193-194 | Position |  | 14 | 156 -159 |
| Branch 1 | Red | - | CTA | AG | C,C,C | CC | G | A | GA------------------ | TG | T | AAC | CT | Sample-ID | DBL_239 | A | ATAT |
|  | Purple | C | CTA | AG | C,C,C | CC | G | A | GA------------------ | TG | T | AAC | CT |  | DBL_290 | A | ---- |
|  | Orange | - | TCT | TA | A,T,T | AA | A | C | TTCCCTAAGAAATGCTAC | AT | A | TTT | TA |  | DBL_831 | C | ATAT |
|  |  |  |  |  |  |  |  |  |  |  |  |  |  |  | DBL_997 | A | ATAT |
| Branch 2 | Position | 159 | 117-181 | 184-190 | 195-199 | 200 to 205 | 207 to 216 | 217 to 219 | 221 to 222 | 224 to 228 | 229 to 236 | 364 |  |  | DBL_690 | A | ATAT |
|  | Yellow | G | GAAAG | TTAAAAA | AGTCA | ------ | ---------- | CTT | CG | GGTGA | CTTTTAGA | A |  |  | GAB_723 | A | ATAT |
|  | Green | A | GAATA | AAAAATA | AATAT | GTAGCA | TTCTTAGGGA | ATG | TG | TTTTA | TATTAGAA | - |  | | | | |
|  | Blue | C | ATTTA | TATTTTT | TTCAT | CCAACA | CTAGTCGGTA | TAG | TT | TGGTT | ------- | A |  | | | | |
| ***Plasmodium vivax* (18S rRNA gene)** | | | | | | | | | | | | | | | | | |
|  | Position | 40, 186 | 48 | 99 | 104, 224,234 | 128,222,235 | 138-142 | 146,303 | 150,306 | 158-159 | 161-164 | 172-173 | 175-176, 188,219 | 190-194 | 202, 279 | 204,216 | 251 |
| Branch 1 | Green | A | C | C | A | T | ----- | C | G | GA | TTTA | AA | T | TAT-T | - | G | CG |
|  | Yellow | A | C | C | A | T | ----- | C | G | GA | TTTA | AA | T | TAT-T | - | G | CG |
|  | Blue | A | C | T | G,G,A | A,G,C | AGACT | C | A,A | AG | TTTA | AG | T | AACCT | T,A | G | AA |
|  | Brown | A | C | C | A | T | ----- | C | G | GA | TTTA | AA | T | TAT-T | - | G | CG |
|  | Black | G,G | T | C | A | T | ----- | T,A | G | GT | AAAC | GA | AG,A,C | AGT-C | - | T,A | CG |
|  |  |  |  |  |  |  |  |  |  |  |  |  |  |  |  |  |  |
|  | Position | 119, 143 | 122,148, 200 | 134,177,190,283 | 145-146, 160-161 | 150,291 | 172-175 |  | | | | | | | | | |
| Branch 2 | Red | G | T, T,T | A,A,A,A | CT, CT | C,C | GTTT |  | | | | | | | | | |
|  | Purple | A | C,A,C | C,T,G,G | TA, AA | T,T | TAAA |  | | | | | | | | | |
